# PyHFO: lightweight deep learning-powered end-to-end high-frequency oscillations analysis application

**DOI:** 10.1088/1741-2552/ad4916

**Published:** 2024-05-28

**Authors:** Yipeng Zhang, Lawrence Liu, Yuanyi Ding, Xin Chen, Tonmoy Monsoor, Atsuro Daida, Shingo Oana, Shaun Hussain, Raman Sankar, Aria Fallah, Cesar Santana-Gomez, Jerome Engel, Richard J Staba, William Speier, Jianguo Zhang, Hiroki Nariai, Vwani Roychowdhury

**Affiliations:** 1 Department of Electrical and Computer Engineering, University of California, Los Angeles, CA, United States of America; 2 Division of Pediatric Neurology, Department of Pediatrics, UCLA Mattel Children’s Hospital, David Geffen School of Medicine, Los Angeles, CA, United States of America; 3 Department of Neurosurgery, UCLA Medical Center, David Geffen School of Medicine, Los Angeles, CA, United States of America; 4 Department of Neurology, UCLA Medical Center, David Geffen School of Medicine, Los Angeles, CA 90095, United States of America; 5 Department of Neurobiology, University of California, Los Angeles, CA, United States of America; 6 Department of Psychiatry and Biobehavioral Sciences, University of California, Los Angeles, CA, United States of America; 7 Department of Radiological Sciences, University of California, Los Angeles, Los Angeles, CA, United States of America; 8 Department of Bioengineering, University of California, Los Angeles, Los Angeles, CA, United States of America; 9 Department of Computer Science and Engineering, Southern University of Science and Technology, Shenzhen, People’s Republic of China

**Keywords:** convolutional neural networks, neurophysiology, high-frequency oscillations

## Abstract

*Objective*. This study aims to develop and validate an end-to-end software platform, PyHFO, that streamlines the application of deep learning (DL) methodologies in detecting neurophysiological biomarkers for epileptogenic zones from EEG recordings. *Approach*. We introduced PyHFO, which enables time-efficient high-frequency oscillation (HFO) detection algorithms like short-term energy and Montreal Neurological Institute and Hospital detectors. It incorporates DL models for artifact and HFO with spike classification, designed to operate efficiently on standard computer hardware. *Main results*. The validation of PyHFO was conducted on three separate datasets: the first comprised solely of grid/strip electrodes, the second a combination of grid/strip and depth electrodes, and the third derived from rodent studies, which sampled the neocortex and hippocampus using depth electrodes. PyHFO demonstrated an ability to handle datasets efficiently, with optimization techniques enabling it to achieve speeds up to 50 times faster than traditional HFO detection applications. Users have the flexibility to employ our pre-trained DL model or use their EEG data for custom model training. *Significance*. PyHFO successfully bridges the computational challenge faced in applying DL techniques to EEG data analysis in epilepsy studies, presenting a feasible solution for both clinical and research settings. By offering a user-friendly and computationally efficient platform, PyHFO paves the way for broader adoption of advanced EEG data analysis tools in clinical practice and fosters potential for large-scale research collaborations.

## 1. Introduction

Human and animal studies of epilepsy have suggested that intracranially-recorded interictal high-frequency oscillations (HFOs) in EEG signals are a promising spatial neurophysiological biomarker of the epileptogenic zone. Many retrospective studies [1–5] have demonstrated that the removal of brain regions producing HFOs correlates with post-operative seizure freedom. More recently, various studies [6–13] have suggested that HFOs potentially have different mechanistic origins, and hence, only a subset of HFO events -often referred to as pathological HFOs—constitute meaningful biomarkers for epileptic zones, while others of physiological origins might be useful for characterizing, for example, the eloquent cortices [12]. Such further refinements of HFOs include tasks, such as artifact rejection, HFOs with spike-wave discharges (spkHFO) detection, epileptogenic HFO discovery, and physiological HFO detection.

However, translating these research findings into a clinical setting to enhance post-operative seizure-free outcomes poses significant challenges. It requires a multidisciplinary approach involving experts in machine learning, artificial intelligence, neurology, and epileptology to refine and establish the clinical relevance of different types of HFOs and potentially discover more effective biomarkers. Such collaboration necessitates the development of a scalable software platform that enables advanced data analysis, annotation, expert verification, and sharing of patient outcome data in a user-friendly manner.

Within the field of EEG studies, a considerable number of open-source software applications aim to offer visualization tools (e.g. EEGLab [14], EEGnet [15], EPViz [16], and Brainstorm [17]) as well as an array of computational biomarker detection algorithms implemented in various programming languages (e.g. MNE [18], YASA [19], and PyEEG [20]) that collectively allow detection and visualization of EEG biomarkers.

Meanwhile, significant efforts have been devoted to deep learning (DL) for event classification in both scalp EEG [21] and invasive EEG [22] to facilitate EEG decoding [23], artifact rejection [24], and disease detection [25]. However, as these methods grow in complexity, there are dramatically increasing computational costs and greater reliance on the technical expertise of operators. Consequently, there is a considerable scientific and engineering gap between the research on DL-powered EEG analysis tools and the distribution of state-of-the-art DL methods to clinicians’ personal computers for practical application. So far, efforts to bridge this gap have been insufficient.

A similar scenario prevails in HFO studies. RIPPLELAB [26], an open-source Matlab-based software, has facilitated early studies on HFOs, incorporating EEG visualization and mainstream HFO detection algorithms. This software is widely used in several studies across the community [27–32]. Simultaneously, many recent studies leverage DL models to carry out HFO analysis [11, 12, 33]. The research community values open-source HFO-analysis software like RIPPLELAB; however, the absence of an integrated platform for clinicians to employ these DL models hinders the full potential of HFOs and associated biomarkers.

Therefore, a software platform compatible with popular DL frameworks is highly desirable for enabling advanced machine learning and DL tools to automate various steps of HFO refinement and deploy them efficiently, even on moderately powerful machines commonly available to clinicians.

In this paper, we present our initial efforts to develop such an application, addressing three key engineering challenges:

• We developed time-efficient detection algorithms of HFO events, by re-implementing the HFO detectors in Python and significantly reducing the detection run-time by at least 93% in comparison to RIPPLELAB in three datasets
• We addressed the demanding task of integration of DL-based HFO classification by simplifying artifact and spkHFO classification networks introduced in a previous study [11], allowing the DL model to run smoothly on the ‘clinician-grade’ CPUs.
• We built an open-source executable software that integrates both time-efficient HFO detection algorithms and simplified artifact and spkHFO classification networks.

The integration of all of the functionalities, PyHFO, holds great potential for facilitating seamless collaboration and enabling large-scale EEG data analysis.

## 2. Method

**Figure 1.**
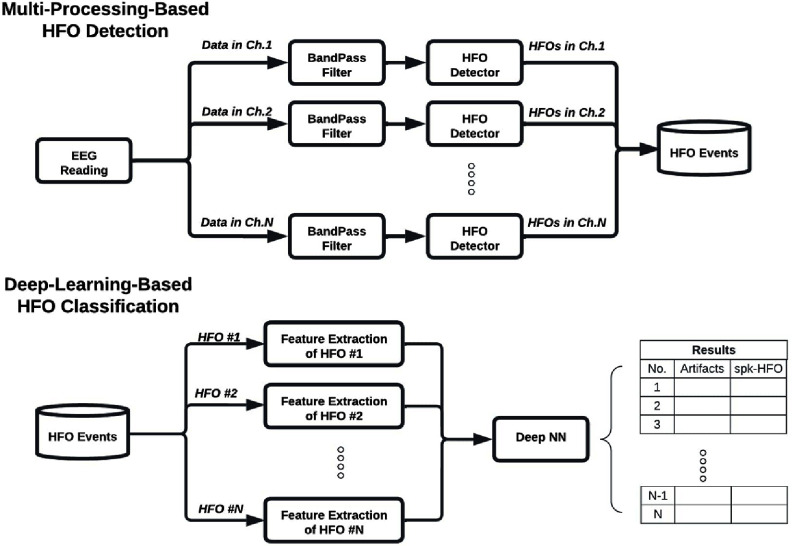
Our study’s overall data processing workflow is shown as a flowchart. We adopted a multi-processing mechanism in both HFO detection and feature extraction for DL networks, which significantly increased the efficiency of the HFO analysis. Specifically, the HFO detectors detected HFO events from the EEG recordings and returned the start and end timestamps of the detected HFO events. For each event detection, the classification pipeline used the start-end information to compute the center and defined a window of width ±285 ms around the center time stamp. These time windows were used to extract EEG segments from the recordings. Then, features computed from the EEG segments were sent to the pretrained DL models for HFO classification. See figure 5 for the overall graphic user interface for PyHFO.

PyHFO is a multi-window graphical user interface (GUI) desktop application specifically designed for the efficient analysis and classification of HFOs. It presents a user-friendly and intuitive interface that caters to both technical and non-technical users, streamlining the process of HFO detection and classification. PyHFO operates through four primary stages upon loading an EEG recording: EEG signal reading, data filtering, HFO detection, and DL-based HFO classification. These stages are detailed in a data flowchart, as seen in figure 1. The output of this pipeline includes detected events based on the implemented detection algorithm, accompanied by annotations of real HFOs, artifacts, and spkHFO, generated using pre-trained DL-based HFO classification models. The specifics of each critical stage are elaborated upon in the subsequent sections.

### 2.1. HFO detection algorithms

In PyHFO, we implemented two automatic HFO detection algorithms into the standalone executable software. We selected the short time energy (STE) [34] and the Montreal Neurological Institute and Hospital detector (MNI) [35] to implement because they are the two primary detection algorithms in the widely used Matlab-based HFO analysis tool, RIPPLELAB, where they have demonstrated success in numerous studies [27–32, 36, 37]. The PyHFO’s modular architecture, coupled with the open-source property of the project, allows for easy integration of alternative HFO detection methods if required. Developers need to adhere to a straightforward interface. Moreover, any added methods will inherently benefit from the established multi-processing paradigm.

We have faithfully replicated the precise parameters and computational implementation of both algorithms from RIPPLELAB in Python. We have put a detailed explanation in the tables B.1 and B.2. However, we have introduced certain modifications. This includes replacing functions, such as the gamma distribution parameter estimation, with the official Scipy’s APIs. Additionally, we have exposed the random seed to the user to ensure the reproducibility of the MNI detector. More importantly, to enhance execution efficiency, we have replaced the for loop with matrix multiplication, particularly in the Gabor wavelet computation. These alterations may result in slightly divergent detection outcomes compared to those obtained from RIPPLELAB. A comprehensive analysis and comparison of these results will be presented in the dedicated analysis section.

### 2.2. HFO detector implementation details

#### 2.2.1. Data reading

PyHFO is designed to accept mainstream EEG data file formats such as the European data format (EDF). Additionally, it can process data in the widely-used NumPy format when users employ the deployed Python package (see section 2.5). In processing an EDF file, raw data is stored in binary format. Upon reading, to convert the digital (raw) values $D_\textrm{raw}$ to the real-world physical voltage *V*, the digital values are calibrated using maximum and minimum physical voltage $V_\textrm{max}, V_\textrm{min}$ and maximum and minimum digital values $D_\textrm{max}, D_\textrm{min}$ The equation used by most EEG data processing tools, such as MNE [18], is given in equation (1). In this equation, *R* is the calibration ratio, defined as the ratio of the difference between the maximum and minimum physical values to the difference between the maximum and minimum digital values, and an offset *O* is defined as the difference between the minimum physical value and the product of the calibration ratio and the minimum digital value.

\begin{equation*} \begin{aligned} V &amp; = R \cdot D_{\text{raw}} + O, \\ \text{where} \quad R &amp; = \frac{V_{\text{max}} - V_{\text{min}}}{D_{\text{max}} - D_{\text{min}}}, \\ O &amp; = V_{\text{min}} - R \cdot D_{\text{min}}. \end{aligned} \end{equation*}

It’s worth noting that RIPPLELAB processes EDF files differently than other mainstream EDF reading tools. Contrastingly, RIPPLELAB performs calibration only through $V = R * D_\textrm{raw}$, with no offset adjustment, resulting in data readings with a DC offset between RIPPLELAB and other EDF reading tools. In our implementation within PyHFO, we have elected to use the more robust calibration equation, equation (1), as the open-sourced Python package MNE.

#### 2.2.2. Signal filtering

**Figure 2.**
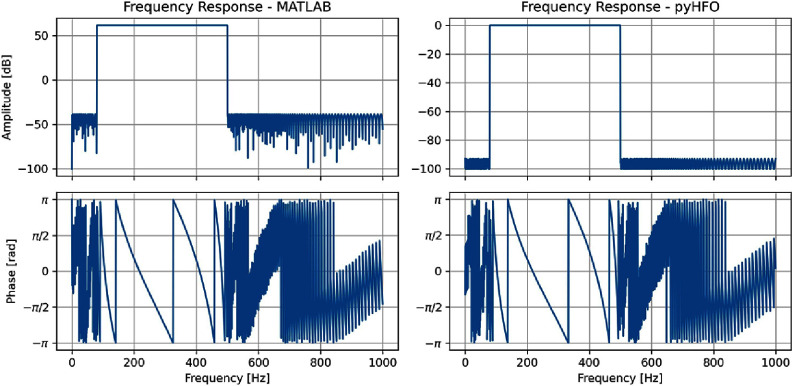
A comparison between Matlab (left) and Scipy (right) regarding filter construction. For the given parameters $F_p = 80$ Hz, $F_s = 500$ Hz, $r_p = 0.5$ dB, and $r_s = 100$ dB, which is specified in RIPPLELAB, Matlab did not precisely match the frequency response to the desired PassBand Ripple and StopBand Attenuation. In contrast, Scipy utilized in our implementation generates a more closely aligned frequency response. Owing to numerical overflow, we use a slightly adjusted StopBand Attenuation value of $r_s = 93$ dB in our PyHFO implementation (*F_p_*: PassBand, *F_s_*: StopBand, *r_p_*: PassBand Ripple, *r_s_*: StopBand Attenuation).

The voltage value read from EDF was then passed through a bandpass filter to extract the signal in the desired frequency domain with the specified ripple and attenuation. The bandpass filter used in the RIPPLELAB was the Chebyshev type II filter; the parameter of this filter consisted of PassBand(*F_p_*), StopBand(*F_s_*), PassBand Ripple(*r_p_*), and StopBand Attenuation(*r_s_*). For constructing such a filter, the order of the filter was first estimated, and then the frequency response was constructed. We noticed that Matlab sometimes could not achieve an exact match to the desired PassBand Ripple and StopBand Attenuation. Therefore, in implementing the PyHFO, we chose to use the filter construction by Scipy as it could produce a more aligned frequency response to the specification. However, since the Scipy could not replicate the filter parameter specified in RIPPLELAB, $F_p = 80$ Hz, $F_s = 500$ Hz, $r_p = 0.5$ dB, and $r_s = 100$ dB due to the numerical overflow, we used the closest number in StopBand Attenuation $r_s = 93$ dB instead in our implementation. We visualized such prementioned phenomena in figure 2.

#### 2.2.3. Multi-processing-based detection framework

To improve the efficiency of our HFO detection pipeline, we leveraged the multi-processing capability of Python. In figure 1, we illustrated how we parallelized the time-consuming steps, namely data filtering and HFO detection, across each channel. Since the computation for each channel was independent, we assigned each channel’s data to different CPU cores to run simultaneously. This approach maximized the use of available CPU cores, leading to a significant reduction in the detection time. To demonstrate the acceleration of our detector’s running speed, we compared the detection times for MNI and STE detectors using our detector and the RIPPLELAB detector on different hardware machines.

### 2.3. Lightweight DL-based HFO classification

We followed the same artifact and spk-HFO classifier design in [11] as it had already shown promising performance against expert annotation. The training data was from HFO detected by STE detector via RIPPELAB in UCLA dataset along with annotation from experts (NH and SH) [11]. However, several limitations prevent them from being directly used in the natural setting: (1) Currently, the HFO analysis is majorly conducted in CPU machines; access to GPU is not very popular in this domain of study. Application of the proposed network in [11] in CPU is time-consuming. (2) Then, the generalization ability of models in [11] cannot be ensured in HFOs detected by other detectors such as MNI. To address (1), we reduced the computational cost of the model by first seeking the smallest information (input size) that can maintain the classification performance and then employing the state-of-the-art neural network pruning technique to reduce the network size. To address limitation (2), we developed a data-augmentation strategy in the neural network training to improve the generalization ability of the model.

#### 2.3.1. DL model training with data augmentation

**Figure 3.**
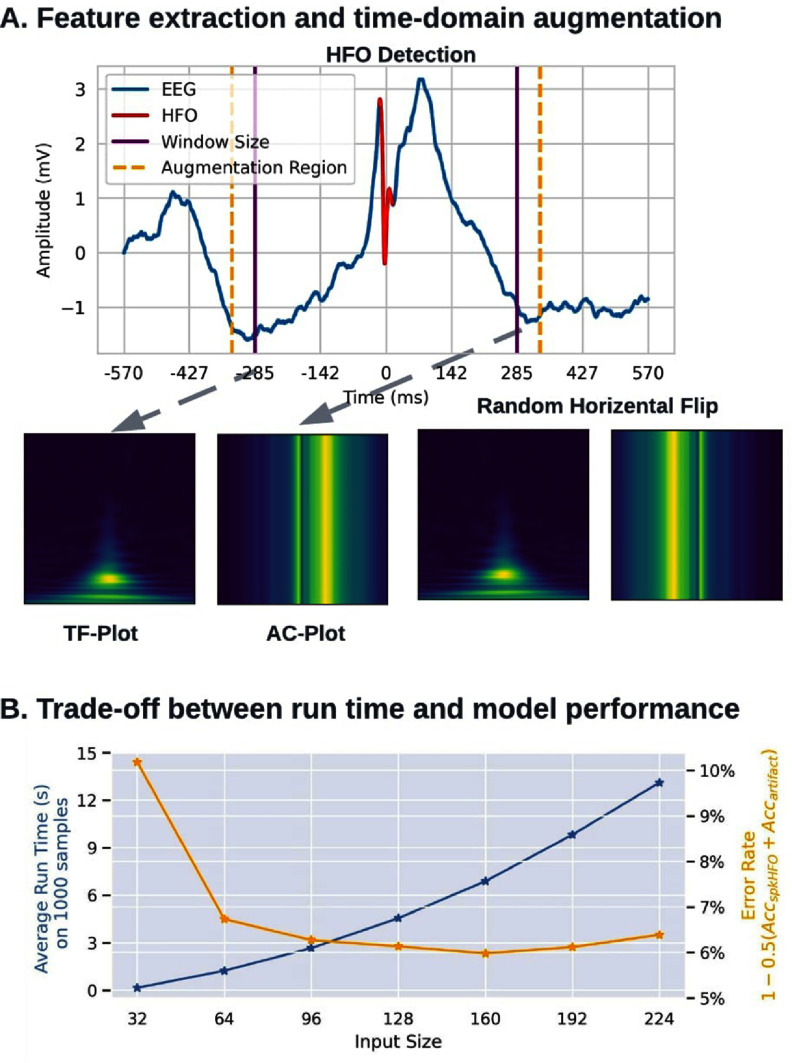
Deep learning network architecture and complexity; (A). The time-domain augmentation consisted of two steps: (1) random time-domain shift, the window of selecting EEG tracing to generate feature (initially center at the middle of the HFO with ±285 ms around it, purple region) could be randomly shifted ±50 ms in the time domain (orange region). (2) the EEG signal was randomly flipped in the time domain. Then, the time-frequency plot (representing 10-290 Hz in the frequency domain and 570 ms in the time domain) and amplitude coding plot with size 128 × 128 are generated. (see figure C.1 for the detailed pipeline and example of how the time-domain augmentation was conducted dynamically during the training). (B). Empirical analysis of the model input size, network performance, and model complexity: While keeping the information resolution (2.18 Hz/pix and 4.46 ms/pix) the same, we trained and evaluated the two classifiers with different input sizes from 32 × 32 (10-80 Hz, ±72 ms) to 224 × 224 (10-500 Hz, ±500 ms), we plotted the error rate of these two classifiers and the average run time with the corresponding input size together. The error rate of these two classifiers was defined as 1–0.5$\left( \text{Acc}_{\text{artifacts}}+\text{Acc}_{\text{spkHFO}}\right)$ in five-fold cross-validation and the average run time (*T*) was the time to predict 1000 samples on CPU using a Linux Machine 10 times, $\frac{1}{10} \sum (T_{\text{artifacts}}+ T_{\text{spkHFO}})$. We chose 128 as the input size because it gave us the best tradeoff between speed and performance. (Acc: accuracy).

Two DL models were trained in PyHFO: the artifact rejection model and the spkHFO classification model. The artifact rejection model classified all events detected from the HFO detector into artifact and real HFO events (the union of spkHFO and non-spkHFO). Meanwhile, the spkHFO classification model classifies the real HFO events into spkHFO and non-spkHFO. The models were evaluated through five-fold cross-validation. The dataset was randomly shuffled and divided into five groups; each group represented the test set of each fold in cross-validation. Within each fold, the remaining 80% of the data was then split into a training set (70% of the whole dataset) and a validation set (10% of the whole dataset). During the training, we used time-domain augmentation to improve the generalization ability of the artifact rejection model and spkHFO classification model. For each event within the training batch, we randomly flipped the EEG signals and randomly shifted the center of the HFO event forward and backward 50 ms, as shown in figures 3 and C.1. Both neural networks were trained for 30 epochs; thus, each data sample was augmented 30 times. In the validation and test sets, augmentation was not applied to ensure that events were represented accurately during evaluation. Both networks were trained with a batch size of 128 using Adam [38] optimizer with a learning rate of 0.0003. The final DL models were selected based on achieving the lowest validation loss across epochs during the training process.

#### 2.3.2. Reduction of the computational cost

**Figure 4.**
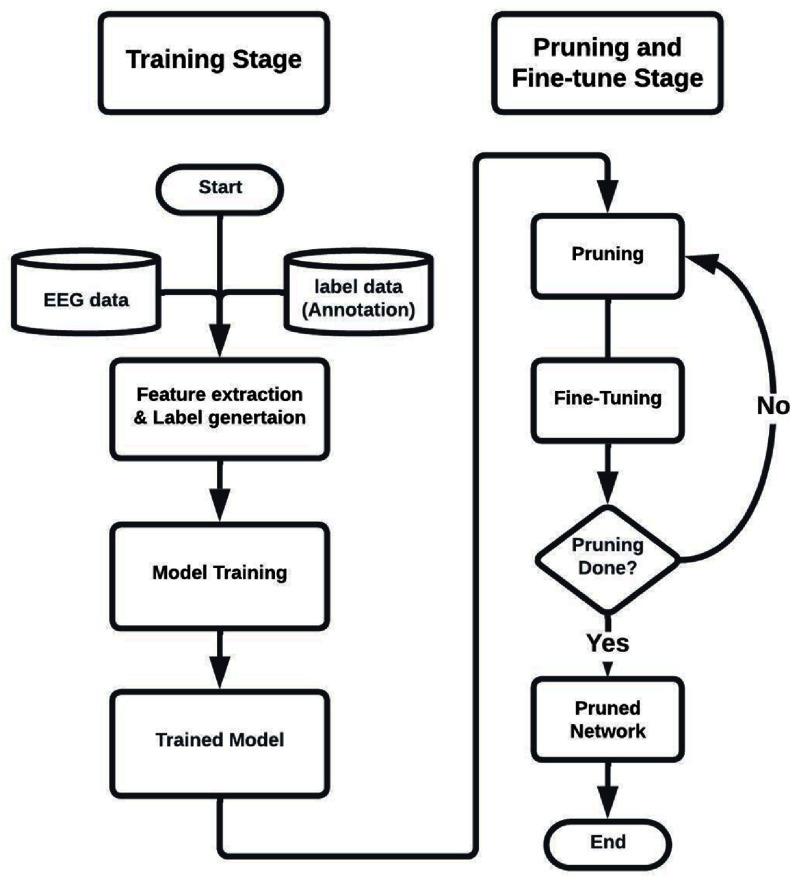
Flowchat of the training and pruning procedure; after the training completes, the pruning and fine-tuning are conducted iteratively to reduce the model size to save computational cost.

The computational cost of a neural network could be measured by the total number of multiply accumulate (MACs) for a fixed number of inputs, which was influenced by the input dimension and the architecture size. To reduce the computational complexity introduced in the input dimension, we first reduced the redundancy in the dimension of the input by only using one time-frequency plot for the artifact detector and concatenation of only the time-frequency plot and amplitude coding plot as input for the spk-HFO classifier. Then, we reduced the input dimension from 224 × 224 to 128 × 128 by only taking 10 to 290 Hz in the frequency domain and ±285 ms of the center of the event in the time domain; these values were chosen by empirical analysis of balancing the computational complexity of the neural network and the classification accuracy (figure 3). Then we simplified the architecture of the artifact and spk-HFO detector, respectively, by pruning the neural network using DepGraph [39] as shown in figure 4. The interactive pruning was conducted for 5000 iterations, and the model was fine-tuned every 250 iterations by five epochs. Finally, we imposed a rule-based filter by treating all HFOs detected in the beginning one second and last one second as artifacts because the beginning and the ending of the recording would lead to artifacts production. The simplified network should run at the best tradeoff between speed and performance in CPU, and we also enabled the use of GPU for users with GPU access on their machine. We evaluated the model complexity by computing MACs using one data sample. Additionally, we measured the average running speed of the model inference on 1000 data samples to get a more straightforward overview of the model complexity.

### 2.4. Framework evaluation

#### 2.4.1. Evaluation patient cohort and intracranial EEG (iEEG) recording

We evaluated the performance of the HFO detectors and classification by using three iEEG datasets.

**UCLA iEEG Dataset (UCLA) [11, 40]:** iEEG data was obtained via grid/strip electrodes using Nihon Kohden Systems (Neurofax 1100A, Irvine, California, USA). The recording was acquired with a digital sampling frequency of 2000 Hz. It contained 19 drug-resistant focal epilepsy subjects. For each subject, separate 10 min EEG segments from slow-wave sleep were selected at least two hours before or after seizures, before anti-seizure medication tapering, and before cortical stimulation mapping, which typically occurred two days after the implant. This dataset contained 19 ten-minute EEG recording segments across 19 patients with 1709 monopolar channels (a median of 94 monopolar channels in each recording). The annotation (Artifact, HFO-with-spike, HFO-without-spike) obtained from expert labeling from a previous study [11] of each STE HFO event was also included in this data (see section 3.3 for detailed annotation statistics).

**Zurich iEEG HFO Dataset (Zurich) [41]:** iEEG data (both grid/strip and depth electrode) was obtained with 4000 Hz digital sampling frequency with an ATLAS recording system (0.5–1000 Hz pass-band, Neuralynx, www.neuralynx.com) and downsampled to 2000 Hz. It contained 20 drug-resistant focal epilepsy subjects. Several runs of five-minute EEG segments of interictal slow-wave sleep were recorded for each subject. We followed the same preprocessing procedure in [41] to create bipolar EEG recordings. This dataset contained 385 five-minute EEG recording segments across 20 patients with 9360 bipolar channels (a median of 23 bipolar channels in each recording).

**UCLA Rodent Dataset (Rodent) [42]:** Rodent iEEG data was obtained with 2000 Hz digital sampling frequency with RHD2000 electrophysiology amplifier chips (pass-band between 0.1 Hz and 1000 Hz). It contained two ten-minute EEG segments from two rodent subjects from the neocortex and hippocampus sampling via depth electrodes. One EEG recording was from a subject with traumatic brain injury (nine channels), and the other one was from a sham-injured control subject (ten channels). Please see the published data [42] for a detailed description of this dataset

#### 2.4.2. Standard protocol approvals, registrations and patient consents

For the UCLA dataset, the institutional review board at UCLA approved using human subjects and waived the need for written informed consent (IRB#18-001 599). All testing was deemed clinically relevant for patient care, and all the retrospective EEG data used for this study were de-identified before data extraction and analysis. This study was not a clinical trial, and it was not registered in any public registry. For the Rodent dataset, All procedures were approved by the University of California Los Angeles Institutional Animal Care and Use Committee (protocol 2000-153-61 A) (for more details, see [43]).

#### 2.4.3. HFO detector parameters

We used the same parameter settings for RIPPLELAB and PyHFO to compare the consistency of their detection results and runtime because PyHFO essentially replicates the detection pipeline of RIPPLELAB. The STE and MNI detectors utilized identical default parameter settings in RIPPLELAB and PyHFO for the UCLA and Zurich datasets. For the Rodent dataset, we applied the suggested parameters for the STE detector as introduced in the original paper [42]. However, since no suggested parameters existed in the MNI detector in [42], we used the default parameter introduced in RIPPLELAB. Tables B.1 and B.2 provides exact parameters used in each dataset.

#### 2.4.4. HFO detector evaluation

To conduct a mathematical evaluation of the detection results between PyHFO and RIPPLELAB, we established a defined representation of events detected by each algorithm. For PyHFO, an event was denoted as $(\textrm{start}_p, \textrm{end}_p)$, indicating the exact time location within the EEG recording. Similarly, for RIPPLELAB, an event was represented as $(\textrm{start}_r, \textrm{end}_r)$. To quantify the degree of overlap between these two sets of events, we introduced the concept of an overlapping ratio, which was defined as $\frac{\min(\textrm{end}_r, \textrm{end}_p)-\max(\textrm{start}_r, \textrm{start}_p)}{\max(\textrm{end}_r, \textrm{end}_p)-\min(\textrm{start}_r, \textrm{start}_p)}$. The resulting value ranged from 0 to 1, with 1 value indicating an exact match. To ensure a fair comparison and avoid double counting, we enforced the condition that an event detected by PyHFO can only matched with a unique event detected by RIPPLELAB. Additionally, the comparison was performed on a channel-by-channel basis, and there was no overlap within events detected by the same detector by the definition of the detecting algorithm. The match of a specific event was defined when the overlapping ratio exceeds a certain threshold, such as 50%. Furthermore, the discrepancy between the two algorithms could be quantified by calculating the ratio of the number of matches to the total number of events detected by RIPPLELAB.

We conducted four experiments to evaluate the success of our detector implementation, assessing the impact of each module independently in the pipeline. For simplicity, we denoted the data reading (Read), filter design (Filter), and detection algorithm (Algo) of RIPPLELAB as Read_*r*_ Filter_*r*_ and Algo_*r*_, where the subscript *r* represents the RIPPLELAB implementation, and we used the subscript *p* to denote the PyHFO implementation. To verify the correctness of our Python implementation, we first extracted the filtered EEG signal from RIPPLELAB. We fed it into our detectors (Read_*r*_ + Filter_*r*_ + Algo_*p*_), comparing the detection overlap with RIPPLELAB (Read_*r*_ + Filter_*r*_ + Algo_*r*_), which we referred to as Exp1. Since we replicated the logic of the two detectors, i.e. Algo_*r*_ = Algo_*p*_, we expected almost 100% matching between them. To assess the impact of the data reading, we conducted Exp2, replicating the frequency and phase response from RIPPLELAB in Python (Read_*p*_ + Filter_*r*_ + Algo_*p*_). For Exp3, we evaluated the effect of the filter design by feeding the EEG signal read by RIPPLELAB into our Python pipeline (Read_*r*_ + Filter_*p*_ + Algo_*p*_). Finally, we compared the complete implementation of PyHFO (Read_*p*_ + Filter_*p*_ + Algo_*p*_) with the RIPPLELAB implementation (Read_*r*_ + Filter_*r*_ + Algo_*r*_). We conducted all experiments on our evaluation cohort and evaluated the implemented STE and MNI, respectively. For each experiment, we compared the detected HFOs with those detected from RIPPLELAB on the total number of HFOs, number of exact match HFOs, and the number of at least 50% overlap, evaluating the discrepancies between RIPPLELAB and our implementation in each step of the data processing. By evaluating the effect of each module independently, we were able to demonstrate the success of our detector implementation, providing a comprehensive assessment of the performance of our PyHFO implementation.

#### 2.4.5. DL-based neural network evaluation

The performance of our trained artifact and spk-HFO detectors was evaluated by comparing the results with the label. We adopted standard metrics for machine learning classification tasks to assess the model’s performance, including $\text{Precision} = \frac{\text{TP}}{\text{TP} + \text{FP}} $, $\text{Recall} = \frac{\text{TP}}{\text{TP} + \text{FN}} $, $\text{Accuracy(Acc)} = \frac{\text{TP} + \text{TN}}{\text{TP+TN+FP+FN}} $, and $\text{F1-score} = 2 \times \frac{\text{Precision} \times \text{Recall}}{\text{Precision} + \text{Recall}}$, where TP represents True Positives, FP represents False Positives, FN represents, and TN represents. Given that the model was trained using five-fold cross-validation on STE HFOs, the reported metric values were the mean results of the five-fold cross-validation (on different test sets) with a 95% confidence interval. To evaluate the model performance on the MNI HFOs, experts annotated MNI HFOs from representative patients. Models trained in five-fold cross-validation were used to predict all events. The reported metric was thus the mean of the metrics from five models, again with a 95% confidence interval.

#### 2.4.6. Runtime analysis

We conducted our run time analysis on a Linux machine, a macOS machine, and a Windows machine. The Linux machine had an AMD Ryzen Threadripper 2950X 32-core processor; the Windows machine had an Intel i9-13 900 K 24-core processor; and the macOS machine had an Apple M1 Pro 8-core processor. For timing the HFO detectors in RIPPLELAB, we followed the modification of RIPPLELAB in [11] to run the Matlab-based detector and report the runtime for detecting STE and MNI HFOs in the Linux machine. For PyHFO, we ran the detector with the same parameters as in RIPPLELAB and reported the runtime by using single-core (*n*-jobs = 1) and multi-core (*n*-jobs = 32 for Linux, *n*-jobs = 8 for Windows and Mac machines). see tables B.1 and B.2 for exact parameter setting in both detectors. For benchmarking the DL models, we used DL models to predict 1000 samples ten times to get the mean and 95% confidence interval of the run time in different machines with PyTorch default setting, and we also reported the inference time on an Nvidia Titan RTX GPU for reference.

### 2.5. Software overview

**Figure 5.**
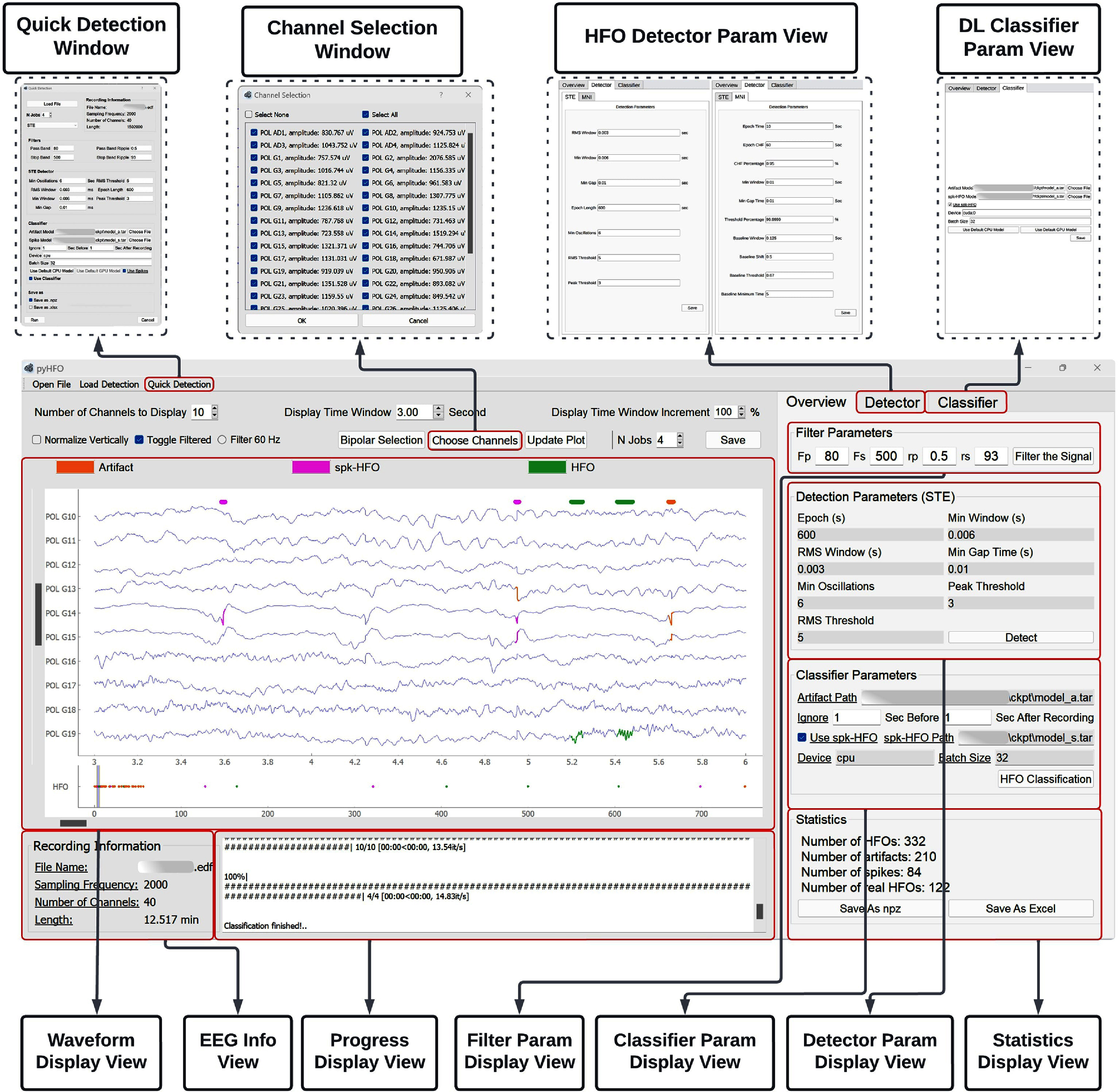
The multi-window overview of the PyHFO application, outlining the user interaction process. For a more detailed explanation, users should consult the PyHFO project page (see section 2.5). In summary, the process is as follows: load EEG: the user can select and load a recording (EDF or BrainVision data format) for EEG data analysis. EEG information and the EEG waveform are then displayed in the ‘EEG info view’ and ‘Waveform Display View’, respectively. HFO detection: users can specify filter parameters in the ‘Filter Param Display View’, select the HFO detector and its parameters in the ‘HFO Detector Param View’, and click ‘detect’ to start HFO detection. The progress is displayed in the ‘Progress Display View’, and detected HFOs are shown in the ‘Waveform Display View’. DL-based HFO classification: users can select a pre-trained network or use the pre-installed models in PyHFO from the ‘DL-Classifier Param View’. After clicking ‘HFO Classification’, a progress bar appears in the ‘Progress Display View’, and once the process completes, classified HFOs are marked in the ‘Waveform Display View’. Results can be exported in Excel or NPZ formats. To simplify the process, users can also use the ‘Quick Detection Window’ to specify all parameters for the whole pipeline, bypassing GUI interaction.

The software version PyHFO is a multi-window GUI developed in PyQt. It is intended to be a user-friendly and intuitive tool that users with technical and non-technical backgrounds can use to detect and classify HFOs time-efficiently. PyHFO has been released under Academic Licenses (Licenses for Sharing Software Code Non-commercially, UCLA TDG). The GUI interface was implemented in PyQt version 5.15 to make it compatible with hardware platforms such as macOS, Linux, and Windows. The HFO detectors were implemented in Python 3.9.0, and the DL-based detector was implemented in PyTorch 1.6. We chose Python as the programming platform because it is widely used in large-scale data analysis and DL in the medical image field. The completed procedure for detecting and analyzing HFOs through PyHFO, consists of several steps briefly discussed in figure 1. We here presented the GUI of the software in figures 5 and E.1. After setting the parameters of different detectors and DL-based classifier, the original EEG signal will be displayed in the main visualization window, and HFO with different attributes (artifacts, HFO-with-spike, and HFO-without-spike) will be annotated with different colors. The statistics of different kinds of HFOs will be displayed on the summary panel for each channel. All of these statistics can be exported in Excel format for further study by the user.

### 2.6. Data sharing and availability of the methods

Anonymized EEG data (UCLA and Rodent) used in this study are available to the corresponding author upon reasonable request. Public iEEG data (Zurich) can be downloaded from open neuro at https://openneuro.org/datasets/ds003498/versions/1.1.1. The source code, documentation, and executable application of the PyHFO software application are available at https://github.com/roychowdhuryresearch/pyHFO. For technical background users, we also release our multi-processed HFO detector in Python Package Index (PyPI), which can be installed by ***pip install HFODetector*** and DL-based HFO classifiers at https://github.com/roychowdhuryresearch/HFO-Classification/tree/main/Pruning-pipeline so that python users can easily install it.

## 3. Results

### 3.1. HFO detector evaluation

In the UCLA dataset, RIPPLELAB detected detected $12,494$ STE HFOs and $10,392$ MNI HFOs, while PyHFO detected $12,501$ STE HFOs and $10,355$ MNI HFOs. In the Zurich dataset, RIPPLELAB detected $31,744$ STE HFOs and $70,988$ MNI HFOs, while PyHFO detected $31,869$ STE HFOs and $70,538$ MNI HFOs. In the rodent dataset, RIPPLELAB detected 375 STE HFOs and 42 MNI HFOs, while PyHFO detected 378 STE HFOs and 39 MNI HFOs. In table 1, we demonstrated the breakdown performance of each experiment. Specifically, in Exp1, we demonstrated that PyHFO successfully replicated the detection algorithms implemented in RIPPLELAB. The discrepancy in the MNI detector was due to different random seed mechanisms RIPPLELAB and PyHFO used. Controlled variable experiments showed that different data readings (Exp2) and filter (Exp3) do affect the performance of the detector but with a minimal effect of around 3 to 7% difference between RIPPLELAB’s detection and PyHFO. The overall discrepancy was defined as the sum of the number of new HFOs detected by the RIPPLELAB (new-RIPPLELAB) and the number of new HFOs detected by the PyHFO (new-PyHFO) divided by the total number of HFOs detected by the RIPPLELAB. The discrepancies between PyHFO and RIPPLELAB of STE and MNI detector were 10% and 14%, respectively.

**Table 1.** Event comparison of differences between RIPPLELAB and PyHFO implementations in UCLA, Zurich, and Rodent dataset. The new-RIPPLELAB row represents the number of new events detected by RIPPLELAB, and the new-PyHFO row represents the number of new events detected by PyHFO for the specific experiment we conducted when the agreement of two events is defined as the 50% overlap of two events. Please note that the difference between 90% overlap and 50% overlap is minimal, amounting to no more than 0.2% $\left(\frac{n_{\text{overlap 50} \%};-n_{\text{overlap 90} \%;}}{n_{\text{overlap 90} \%;}}\right)$. (Exp1: data reading and bandpass filter were from RIPPLELAB, but detection algorithm was from PyHFO; Exp2: data reading was from RIPPLELAB, but bandpass filter and detection algorithm were from PyHFO; Exp3: bandpass filter was from RIPPLELAB, but data reading and detection algorithm were from PyHFO; PyHFO: all data reading, bandpass filter and detection algorithm were from PyHFO).

	No. Events from STE detector					No. Events from MNI detector				
	RIPPLELAB	Exp1	Exp2	Exp3	PyHFO	RIPPLELAB	Exp1	Exp2	Exp3	PyHFO
UCLA										
Total HFO	12 494	12 494	12 421	12 582	12 501	10 392	10 390	10 320	10 422	10 355
Exactly same	—	12 494	11 442	9147	8643	—	10 368	10 035	7729	7487
90% overlap	—	12 494	12 192	12 126	11 876	—	10 390	10 136	9828	9612
50% overlap	—	12 494	12 192	12 144	11 892	—	10 390	10 136	9835	9619
new-RIPPLELAB	—	0	302	350	602	—	2	256	557	773
new-PyHFO	—	0	229	438	609	—	0	184	587	736
Zurich										
Total HFO	31 744	31 744	31 527	32 089	31 869	70 988	70 538	69 760	71 994	71 067
Exactly same	—	31 744	28 330	22 372	20 656	—	70 496	68 361	52 158	50 939
90% overlap	—	31 744	30 667	30 703	29 775	—	70 532	68 860	67 183	65 739
50% overlap	—	31 744	30 667	30 740	29 811	—	70 532	68 862	67 277	65 834
new-RIPPLELAB	—	0	1077	1004	1933	—	456	2126	3711	5154
new-PyHFO	—	0	860	1349	2059	—	6	898	4717	5233
Rodent										
Total HFO	375	375	375	378	378	42	42	39	42	39
Exactly same	—	375	374	324	325	—	42	39	34	31
90% overlap	—	375	375	370	370	—	42	39	42	39
50% overlap	—	375	375	370	370	—	42	39	42	39
new-RIPPLELAB	—	0	0	5	5	—	0	3	0	3
new-PyHFO	—	0	0	8	8	—	0	0	0	0

As highlighted in section 2.2, the implementation within PyHFO closely adheres to prevailing methods for data reading. Additionally, it provides a more accurate representation of the input parameters utilized in the construction of the bandpass filter. Consequently, PyHFO’s methodology exhibits greater implementation accuracy compared to most mainstream publicly released software.

### 3.2. HFO detector runtime comparison

Table 2 presented a runtime comparison between PyHFO and its Matlab-based counterpart (RIPPLELAB) across various hardware specifications on UCLA, Zurich, and Rodent datasets. To save computational resources, we reported only the runtime of RIPPLELAB on the Linux machine. Since different datasets had different numbers of recordings and lengths, we normalized the runtime to measure the detection speed: how many seconds the detector will take to process one minute of recording in one channel (120k data samples at sampling frequency = 2000 Hz). We also put the rough total time processing each dataset in parentheses (see table D.2 for detailed runtime in minutes). When comparing the detection speed of HFO detection by using the same detection parameters, PyHFO significantly outperformed RIPPLELAB in both single-core (*n* = 1) and multi-core (*n* $ > $ 1) configurations, as detailed in 2.4.6 for hardware setup specifications. For the UCLA dataset, the detection speed of PyHFO could be at least 50 times faster (1.309 seconds/channel/minute for RIPPLELAB and 0.018 seconds/channel/minute for PyHFO on STE detector and 19.82 seconds/channel/minute for RIPPLELAB and 0.292 seconds/channel/minute for PyHFO on MNI detector). PyHFO in the UCLA dataset (median of 94 channels per recording) can utilize the multi-processing (when *n* = 32) better than that in the Zurich dataset (median of 23 channels per recording) and Rodent dataset (median of 9.5 channels per recording). Nonetheless, the PyHFO ran at least 15 times faster than RIPPLELAB in these three datasets. Even when operating with a single core, PyHFO still offers at least a six-times improvement in speed. Compared to STE, the MNI detector’s longer runtime is due to an iterative procedure within its computational pipeline. It took days for RIPPLELAB to detect MNI HFOs for a fairly large dataset (4 days for the UCLA dataset and 6.6 days for the Zurich dataset), which blocked feasibility for large-scale HFO analysis in the community. However, using the PyHFO under a multi-core setting, the runtime could be significantly reduced to within six hours.

**Table 2.** Comparative analysis of runtime (measured in runtime (seconds)/channel/recording minutes) in RIPPLELAB and PyHFO: Detection of all events from UCLA, Zurich, and Rodent dataset. We put the roughly total time of detection into parentheses. When *n* $ > $ 1 represents when PyHFO runs in a multi-core setup, *n*-jobs = 32 for the Linux machine and *n*-jobs = 8 for macOS and Windows machines. The best performance in each machine and dataset was highlighted in bold. Abbreviation: d: day(s), h: hour(s), m: minute(s), s: second(s).

	Linux		Windows		macOS	
	STE	MNI	STE	MNI	STE	MNI
UCLA (19 ten-minute recordings, 1709 channels)						
RIPPLELAB	1.309 (≈5.2 h)	19.82 (≈4d)	—	—	—	—
PyHFO(*n*=1)	0.201 (≈1.0 h)	3.410 (≈16 h)	0.121 (≈0.5 h)	3.277 (≈16 h)	0.13 (≈0.5 h)	2.316 (≈11 h)
PyHFO(*n* $ > $ 1)	**0.018 (≈5.2 m)**	**0.292 (≈1.4 h)**	**0.031 (≈9.0 m)**	**0.398 (≈1.9 h)**	**0.026 (≈7.7 m)**	**0.401 (≈1.9 h)**
Zurich (385 five-minute recordings, 9360 channels)						
RIPPLELAB	0.600 (≈7.8 h)	12.30 (≈6.6d)	—	—	—	—
PyHFO(*n*=1)	0.165 (≈2.2 h)	3.097 (≈1.6d)	0.089 (≈1.2 h)	2.014 (≈1d)	0.136 (≈1.8 h)	1.856 (≈1d)
PyHFO(*n* $ > $ 1)	**0.029 (≈22 m)**	**0.415 (≈5.5 h)**	**0.028 (≈22 m)**	**0.411 (≈5.3 h)**	**0.037 (≈28 m)**	**0.304 (≈4.0 h)**
Rodent (2 ten-minute recordings, 19 channels)						
RIPPLELAB	0.473 (≈1.5 m)	15.72 (≈50 m)	—	—	—	—
PyHFO(*n*=1)	0.121 (≈23 s)	4.399 (≈13.9 m)	0.084 (≈16 s)	2.725 (≈8.6 m)	0.111 (≈21 s)	1.983 (≈6.3 m)
PyHFO(*n* $ > $ 1)	**0.032 (≈6 s)**	**1.042 (≈3.3 m)**	**0.041 (≈8 s)**	**0.795 (≈2.5 m)**	**0.047 (≈9 s)**	**0.631 (≈2.0 m)**

### 3.3. HFO event annotation

The HFO annotation was conducted on STE HFO events from the UCLA dataset (*n* = 12 494). Two experts (NH and SH) annotated HFO events into one of the three classes: artifact, HFO-with-spike (spkHFO), and HFO-without-spike (non-spkHFO). The inter-rater reliability of these two expert annotators was measured by the Cohen kappa score (kappa = 0.96 for labeling artifact, 0.85 for labeling HFO-with-spikes). The evaluation procedure was reported in [11]. This annotation yielded 6294 HFOs with spikes (spkHFO), 3459 HFOs without spikes (non-spkHFO), and 2741 artifacts. To ensure the DL models trained from the STE detector also generalize well in HFO events detected by the MNI detector, an expert (HN) annotated MNI HFOs into the artifact, spkHFO, non-spkHFO from representative subjects (3 subjects, *n* = 758, included 416 artifacts, 312 spkHFOs, and 30 non-spkHFO) and the performance metric of the model against the expert annotation was reported.

### 3.4. Machine learning algorithm against expert labeling

In five-fold cross-validation, for STE HFOs (19 subjects, *n* = 12 494), the model achieved an accuracy of 98.6% and 89.1% for classifying artifacts and HFO with spikes, respectively, as shown in table 3. This performance is almost the same as that was [11] (artifacts: 98.8%, spkHFO 89.1%) but with much lower MACs and runtime when we classified HFOs in CPUs in table 4. More importantly, the trained model using STE HFOs could successfully classify the MNI HFOs, demonstrating the success of the data augmentation and generalization-ability of the model, which enables these two DL models to be used in natural settings. The excellent performance across detectors also demonstrates the morphological similarity between the spkHFO in MNI and STE detectors.

**Table 3.** Performance analysis using five-fold cross-validation: mean of accuracy, f1-core, recall, and precision on the test set in five-fold cross-validation with 95% confidence interval versus expert labeling.

	STE		MNI	
	Artifacts	spkHFO	Artifacts	spkHFO
Accuracy	98.56 ± 0.19	89.18 ± 0.82	98.72 ± 0.64	89.79 ± 3.12
F1-score	99.08 ± 0.13	91.40 ± 0.60	90.16 ± 4.88	94.14 ± 1.93
Recall	99.18 ± 0.27	90.78 ± 1.91	94.79 ± 2.72	90.85 ± 4.15
Precision	98.98 ± 0.29	92.07 ± 2.11	100.0 ± 0.00	97.75 ± 0.69

**Table 4.** Comparative analysis of computational costs across models: MACs and model run-time (in seconds) for GPU, Linux, mac OS, and Windows (CNN was the model design in [11]; Dim. Optim. was the run-time after input dimension optimization; Pruning was the model runtime after dimension optimization and pruning). The best performance was highlighted in bold.

	MACs	GPU	Linux	macOS	Windows
Artifact rejection					
CNN [11]	1.82G	0.14 ± 0.10	9.78 ± 0.20	20.28 ± 1.89	15.68 ± 0.10
Dim. Optim.	568.94 M	0.04 ± 0.01	3.52 ± 0.13	4.13 ± 0.02	6.93 ± 0.04
Pruning	**146.05 M**	**0.02** ± 0.00	**1.53** ± 0.05	**1.68** ± 0.01	**2.88** ± 0.01
spkHFO classification					
CNN [11]	1.82G	0.14 ± 0.11	9.84 ± 0.25	19.43 ± 1.16	16.01 ± 0.74
Dim. Optim.	581.58 M	0.17 ± 0.01	3.67 ± 0.11	4.14 ± 0.02	7.49 ± 0.02
Pruning	**152.37 M**	**0.04** ± 0.01	**1.56** ± 0.05	**1.71** ± 0.02	**3.42** ± 0.03

### 3.5. Neural network complexity comparison

In table 4, we compare the MACs on a single data sample as input and runtime of inference 1000 data samples using GPU and CPUs between state-of-the-art and PyHFO. We reported the performance metric of spk-HFO and artifact classifier, respectively. By computing the MACs, the classifiers in PyHFO are more computationally efficient than the models proposed in [11], which provide theoretical support for later empirical experiments. Furthermore, even though both classifiers from [11] and PyHFO run at comparable speeds in GPU, the artifacts classifier in PyHFO runs at least 4 times faster than its counterpart, and PyHFO’s spkHFO detector runs three times faster in CPUs. As another ablation study, we blindly pruned the published network using the same pruning and fine-tuning parameters but without input dimension optimization. Even though the performance of the pruned model was still comparable, MACs were still around 500 M which is much higher than our approach.

## 4. Discussion

Our work was implemented based on strong clinical motivation. Prior observational studies [5, 40] and a clinical trial [4] have shown issues with time constraints in HFO analysis in clinical settings. The clinical use of HFOs detection sites during epilepsy surgery planning requires a fast, reliable, and user-friendly application of HFO detection. It also needs to simulate human experts’ judgment to complement the entire process, including the detection and classification of HFOs. Our platform incorporated such capacities and has capacities to use multiple HFO detection methods and also has classifiers, including artifact rejection and HFOs with spikes vs. without spikes. Additionally, this system is portable, allowing any physician or researcher with a laptop to utilize DL-based algorithms in various settings, such as the epilepsy monitoring unit or the operating room. This capability has the potential to facilitate clinical trials. While developing our PyHFO application, we demonstrated that our HFO detection algorithm is comparable with another open-source work, RIPPLELAB. We comprehensively tested our HFO detection algorithms on three datasets: UCLA (grids/strips), Zurich (grids/strips and depth electrodes), and Rodent dataset. While we implemented the Python version of this HFO analysis application, EEG data reading and input format were deployed using the Python package. We followed the same EEG reading calibration as MNE [18], while in RIPPLELAB, the calibration was only done by voltage, without the offset adjustment. Furthermore, there were slight differences in the data filtering implementation. We chose to use the filter construction by Scipy as it can produce a more accurate frequency response. Our study reported minor differences in HFO detection numbers between RIPPLELAB and PyHFO, and we concluded that those are based on differences in data reading and filtering implementation between Matlab and Python. We fully credit RIPPLELAB for developing the pioneering, user-friendly, MATLAB-based HFO analysis software. This foundational effort greatly informed our Python-based platform, and we also proved that our Python-based implementation is accurate based on engineering aspects.

We combined multiple methods to decrease the run time of the whole pipeline. We utilized the multi-processing feature of Python, employed vectorization implementation in wavelet computation, optimized the neural network’s input size and pruned the neural network architecture. We also developed a data-augmentation strategy in the neural network training to improve the generalization ability of the model. We demonstrated that with the use of our application, the run-time was at least 15 times faster in STE detection and MNI detection compared to RIPPLELAB. We also achieved high performance in classifying artifacts and HFOs with spikes (98.6% and 89.1%, respectively, on five-fold cross-validation and 98.7% and 89.8%, respectively, on an independently annotated test set).

There are several limitations to our study. Our study did not validate our detected HFOs against clinical outcomes, such as postoperative seizure outcomes. Rather, we aimed to establish the engineering validity of our detection algorithms using grid/strip, SEEG, and rat EEG data. The generalizability of our application is still considered limited as it stands, based on a relatively small number of datasets we tested on. However, our application has the potential to expand its capacity. Additionally, there were some interesting features we did not include in this current implementation. For example, (1) the current artifact rejection only considered event-level classification but did not consider cross-channel artifacts (HFOs occur simultaneously across many channels); (2) a standalone interface for detecting ripple and fast ripple separately; (3) data reading from more brain recording formats such as BioSemi data format (.bdf).

In the near future, we have plans to incorporate a diverse dataset into our system. We aim to test the system on a larger dataset comprising over 100 subjects, including pediatric and adult patient data acquired through grids/strips and SEEG. We will also be able to validate the detection results against clinical outcomes using larger datasets. The versatility of our application is evident as we strive to incorporate additional detection methods, such as Hilbert [44] or SLL [45]. As we continuously expand the dataset and introduce new functionalities, the algorithm’s performance will progressively improve through training. The invaluable real-time feedback from frontline physicians and researchers will contribute significantly to this iterative process.

## Figures and Tables

**Figure A.1.**
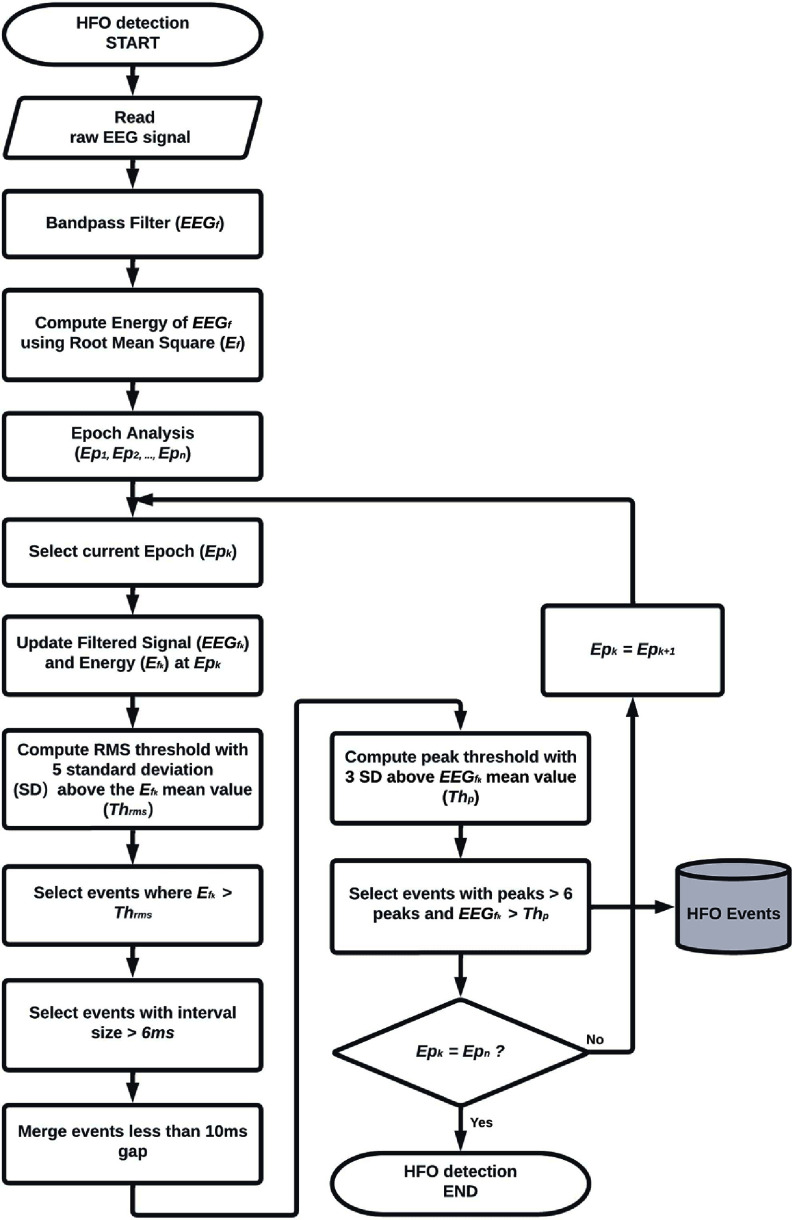
The computational flowchart of the STE HFO detection algorithm.

**Figure A.2.**
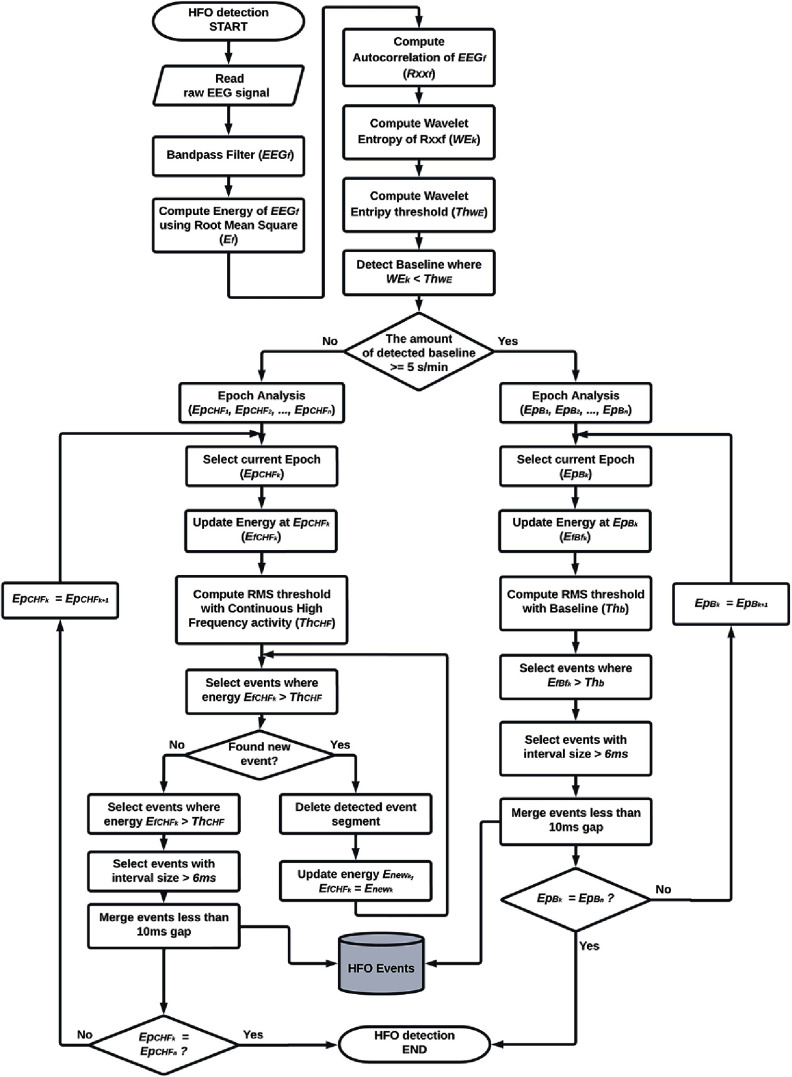
The computational flowchart of the MNI HFO detection algorithm.

**Figure C.1.**
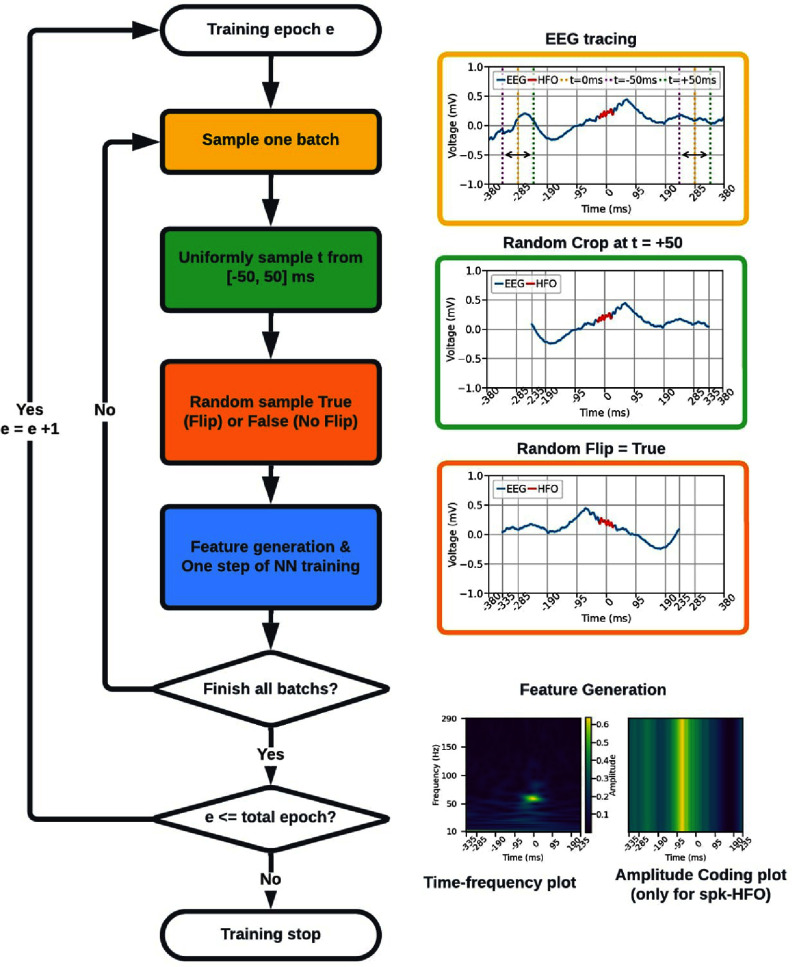
Data augmentation was dynamically conducted during neural network training. Within each training epoch, batches of data were sampled from the dataset. For each data sample (HFO event), a time-domain augmentation shift, t, was uniformly sampled from −50 ms to +50 ms. The region of interest for generating features (centered at the middle of the HFO with a 285 ms window) was then shifted on the time axis to select an EEG tracing; for example, in this figure, when *t* = +50 ms, the window became −235 ms to 335 ms from −285 ms to 285 ms. Subsequently, an integer (0 or 1) was randomly chosen to determine whether the EEG tracing should be flipped in the time domain, with 0 indicating no flipping and 1 indicating flipping. The processed EEG tracing was used to generate two feature images: the time-frequency plot and the amplitude coding plot (the latter only for spk-HFO classification). These images were then fed into the CNN for neural network training.

**Figure D.1.**
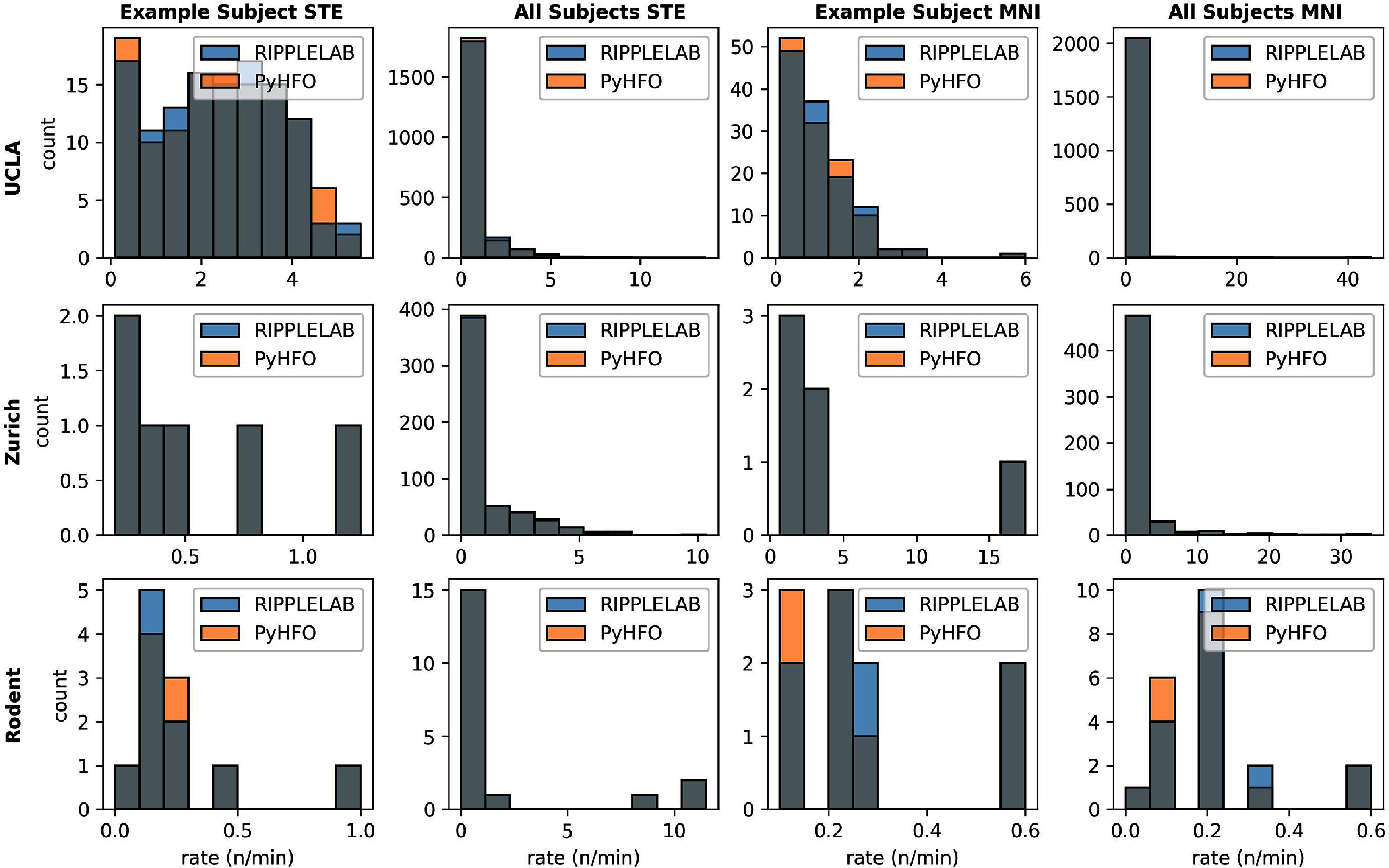
HFO rate comparison across three datasets and two detectors. We calculated the HFO rate, measured as the number of events per minute (n min^−1^), in each channel from each patient and plotted the histogram of the rate in each channel from example patients (column 1 for STE HFOs and column 3 for MNI HFOs) and from all patients (column 2 for STE HFOs and column 4 for MNI HFOs). The gray area of the histogram indicates the detections by both RIPPLELAB and PyHFO. Events only detected by RIPPLELAB are shown in blue, and events only detected by PyHFO are shown in orange.

**Figure D.2.**
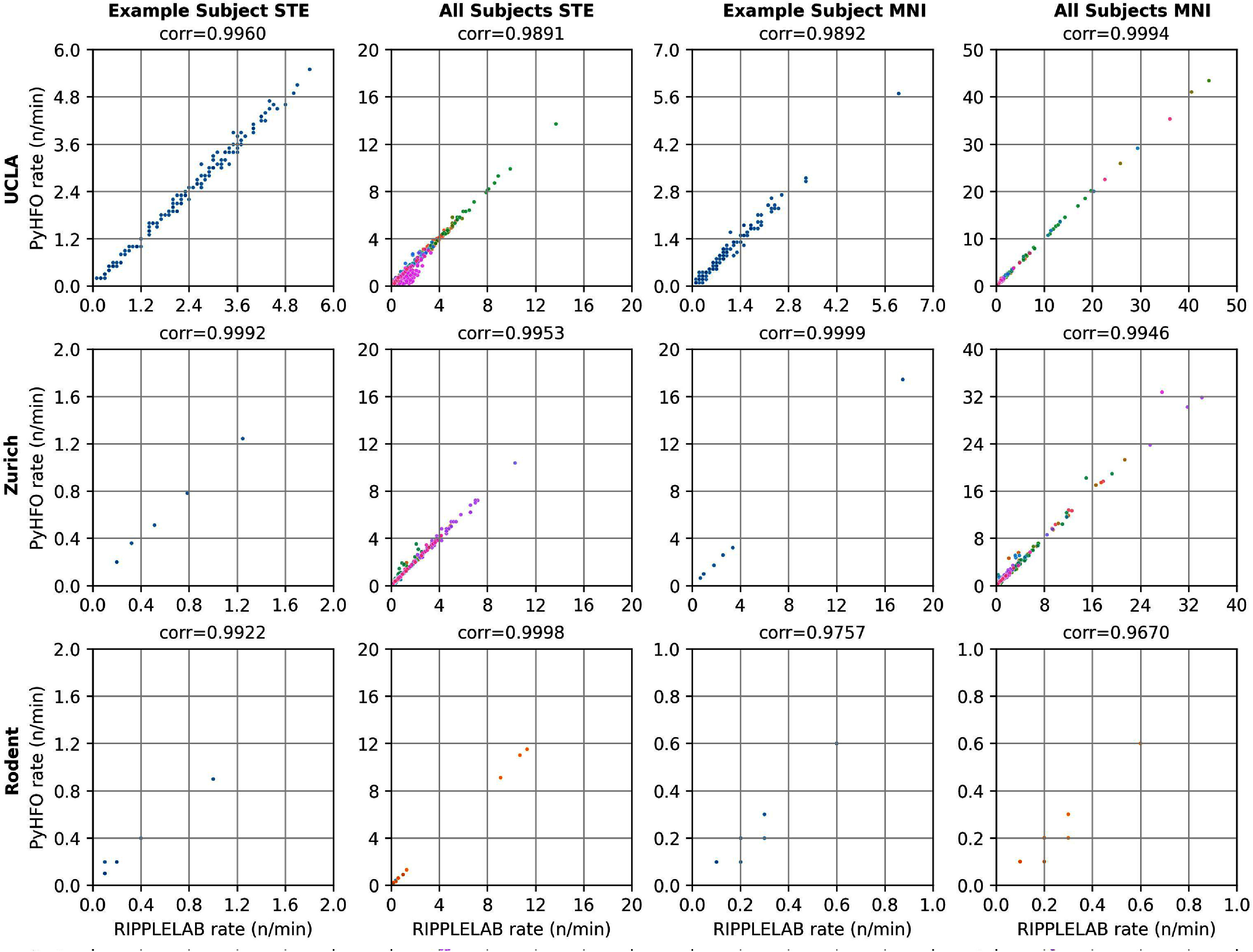
Comparison of HFO rates between RIPPLELAB and PyHFO across three datasets and two detectors. We calculated the HFO rate, expressed as events per minute (n/min), for each channel in each subject and plotted these using scatter plots. The *x*-axis represented RIPPLELAB rates, while the *y*-axis represented PyHFO rates, with each data point corresponding to one channel. The plots were organized into four columns: columns 1 and 3 displayed HFO rates from example subjects (STE and MNI HFOs, respectively), while columns 2 and 4 displayed rates across all subjects, using different colors to represent various subjects. Additionally, the Pearson correlation coefficients, displayed at the top of each subplot, indicate a high degree of correlation, suggesting comparable performance between PyHFO and RIPPLELAB.

**Figure E.1.**
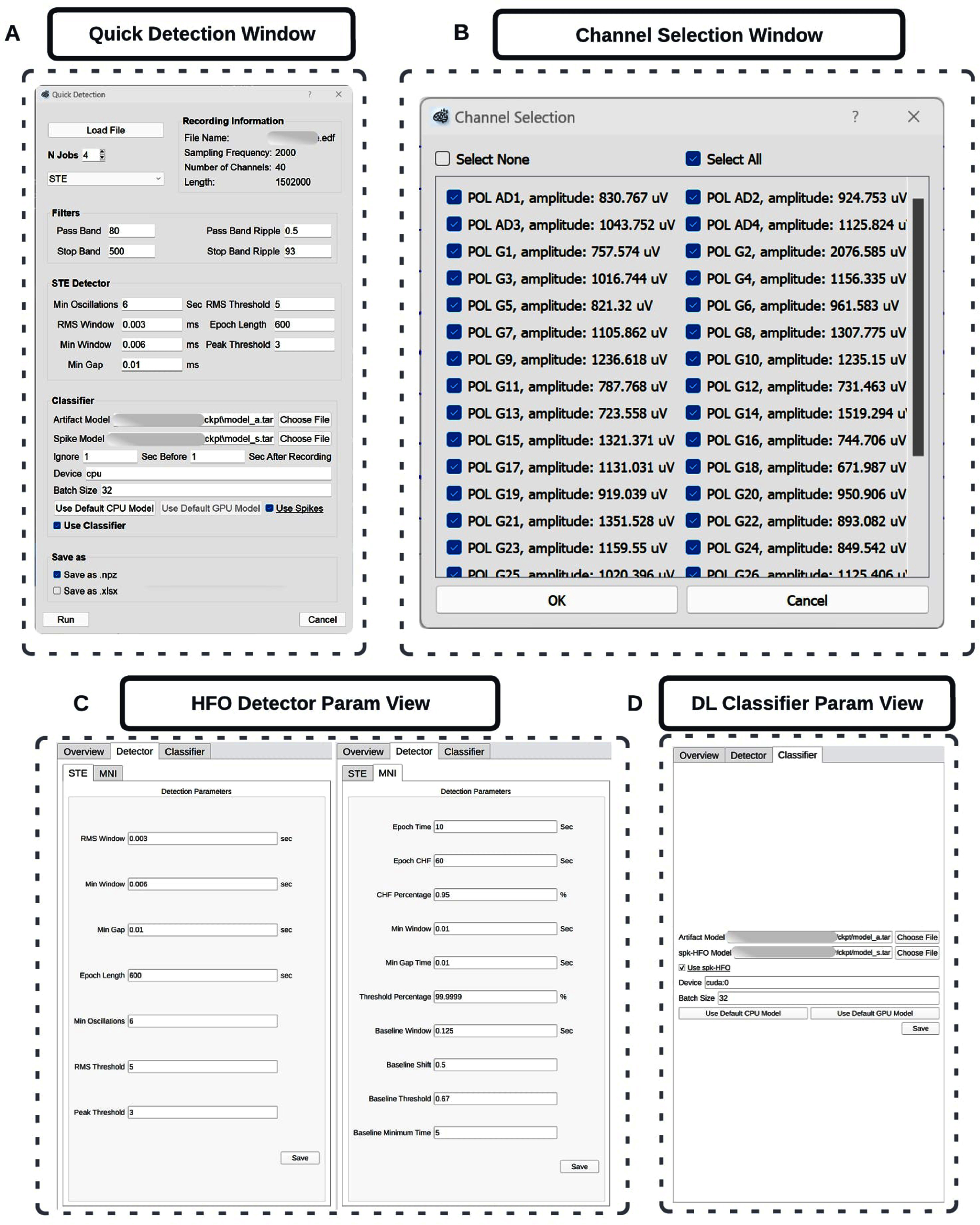
Detailed showcase of each sub-window in PyHFO. A. ‘Quick Detection Window’ is for users to simplify the process by specifying all parameters for the whole pipeline, bypassing GUI interaction. B. ‘Channel Selection Window’ is for users to select desired channels to display in the GUI. C. ‘HFO Detector Param(eter) View’ is for users to specify parameters in STE and MNI detector. D. ‘DL Classifier Param(enter) View’ is for users to specify parameters for deep learning HFO classification model; in this view, the users can specify the pre-trained DL model released within PyHFO or use their own models trained and pruned by the released training pipeline.

## Data Availability

The data cannot be made publicly available upon publication because they contain sensitive personal information. The data that support the findings of this study are available upon reasonable request from the authors.

## Appendix A Details of two detection algorithms

### A.1. STE HFO detection algorithm

The first implemented algorithm, STE, detects HFO events by selecting the energy of the filtered raw EEG signal with an estimated energy threshold for each 10 min epoch. In detail, the EEG signal is processed through a bandpass filter in frequencies between 80 and 500 Hz. The energy of the filtered signal is then computed based on root mean square (RMS) with *N* = 3 ms window. The estimation of the energy threshold is 5 standard deviations (SD) above the overall RMS mean. Finally, all HFO events selected should have a duration of more than 6 ms and contain more than 6 peaks greater than 3 SD above the filtered signal mean value. Figure A.1 demostrated the computational flowchart.

### A.2. Montreal Neurological Institute’s HFO detection algorithm

Another implemented algorithm MNI detector (MNI) was proposed by Zelmann *et al* For this approach, similar to the STE algorithm, the raw EEG signal is also filtered by the bandpass filter and the energy of the filtered signal is then computed using RMS with 2 ms window. A key block for MNI algorithm is the baseline detector, designed to construct the baseline interval. The baseline, defined as EEG segments with no oscillation, is detected by computing the wavelet entropy of the autocorrelation of the filtered signal. For each 125 ms EEG segment with 50% shift, the segment is considered as baseline if the wavelet entropy is greater than the threshold. From the baseline detector, possible HFO events could be detected by selecting energy of the filtered signal using RMS above the energy threshold for each epoch. The energy threshold is computed in two different methods if more than or less than 5 s min^−1^ of the amount of all detected baseline. If a sufficient baseline was found, the energy threshold was selected from RMS values for each 10 s baseline segment at the 99.9999 percentile of its empirical cumulative distribution function. If less baseline was detected, we consider the channel with continuous high-frequency oscillatory activity. The energy threshold is iteratively selected from RMS values for each 60 s segment at the 95 percentile of its empirical cumulative distribution function. The value is found by continuously detecting and removing the highest energy till no more new HFO events are detected. All possible HFO events from the two situations are finally selected with a duration of more than 6 ms. Figure A.2 demostrated the computational flowchart.

## Appendix B HFO detector parameters

We used the same parameter settings for RIPPLELAB and PyHFO to compare the consistency of their detection results and runtime because PyHFO essentially replicated the detection pipeline of RIPPLELAB. The STE and MNI detectors utilized default parameter settings in RIPPLELAB and PyHFO for the UCLA and Zurich datasets. For the Rodent dataset, we applied the suggested parameters for the STE detector as introduced in the original paper [42]. However, since no suggested parameters existed in the MNI detector in [42], we used the default parameter introduced in RIPPLELAB. Tables B.1 and B.2 showed the exact parameters of STE and MNI detectors in each dataset. In these tables, we used the naming of the parameters from PyHFO, but it was easy to find the corresponding parameters from RIPPLELAB.

**Table B.1.** Detailed parameters with units in parenthesis of STE detector used in UCLA, Zurich, and Rodent datasets; filter_freq: frequency band in bandpass filter; rms_window: RMS window time; min_window: minimum time for an HFO; min_gap: minimum distance time between two HFO candidates; min_osc: minimum oscillations per interval; rms_thres: threshold for RMS in standard deviation (SD); epoch_len: duration of determining energy threshold.

	UCLA	Zurich	Rodent
filter_freq (Hz)	[80, 500]	[80, 500]	[80, 500]
rms_window (s)	$3\times 10^{-3}$	3$\times 10^{-3}$	3$\times 10^{-3}$
min_window (s)	6$\times 10^{-3}$	6$\times 10^{-3}$	6$\times 10^{-3}$
min_gap (s)	10*1$\times 10^{-3}$	10*1$\times 10^{-3}$	10*1$\times 10^{-3}$
min_osc (count)	6	6	4
rms_thres (in SD)	5	5	5
peak_thres (in SD)	3	3	2
epoch_len (ms)	600	600	600

**Table B.2.** Detailed parameters with units in parenthesis of MNI detector used in UCLA, Zurich, and Rodent datasets. filter_freq: frequency band in bandpass filter; epo_CHF: continuous high-frequency epoch; per_CHF: continuous high-frequency percentile threshold; min_win: minimum HFO time; min_gap: minimum distance time between two HFO candidates; thrd_perc: threshold percentile; base_seg: baseline window; base_shift: baseline shift window (representing the ratio of the baseline window is shifted); base_thrd: baseline threshold; base_min: baseline minimum time.

	UCLA	Zurich	Rodent
filter_freq (Hz)	[80 500]	[80 500]	[80 500]
epo_CHF (s)	60	60	60
per_CHF (%)	95%	95%	95%
min_win (s)	10$\times 10^{-3}$	10$\times 10^{-3}$	10$\times 10^{-3}$
min_gap (s)	10$\times 10^{-3}$	10$\times 10^{-3}$	10$\times 10^{-3}$
thrd_perc (%)	99.9999%	99.9999%	99.9999%
base_seg (s)	125$\times 10^{-3}$	125$\times 10^{-3}$	125$\times 10^{-3}$
base_shift (0-1)	0.5	0.5	0.5
base_thrd (0-1)	0.67	0.67	0.67
base_min (%)	5	5	5
epoch_time (ms)	10	10	10
seed	0	0	0

## Appendix C Data augmentation

The data augmentation was conducted dynamically with model training; transformations of data samples (HFO event/EEG tracing) could be randomized at each batch, leading to a more robust model. In PyHFO, we trained our models using two augmentation types: time-domain random shifting and time-domain random flipping. Specifically, each data sample (HFO event/EEG tracing) underwent this augmentation process in each training epoch. We trained our models in 30 epochs; thus, we would have 30 variations of each event during the training. Figure C.1 demonstrated this procedure with a detailed example.

## Appendix D More statistics on detection comparison

### D.1. Relevance for clinical decisions

We drew the HFO rates from all detected results from PyHFO and RIPPLELA. We demonstrated the HFO rate (number of events/minutes) for each channel from one example subject and all subjects across all three datasets in term of histograms (figure D.1) and scatter plots (figure D.2) comparison. The high correlation between the HFO rates produced by PyHFO and RIPPLELAB demonstrated that using PyHFO will not affect clinical decisions based on the HFO rate.

### D.2. Discrepancy analysis

Since the table 1 only compared the raw detected events but did not include if the detection results discrepancy was mostly from artifact events or real events. We then used the trained artifact rejection model to predict all the new events from PyHFO and RIPPLELAB. Table D.1 showed classification results for the new events from PyHFO and RIPPLELAB. The accuracy of the artifact rejection model was almost 99% in comparison to the expert labeling; we believed that the trained DL model was good enough to replicate the expert behavior in classifying artifact events and real HFO events. In table D.1, even though the number of artifacts in newly detected events was slightly lower for PyHFO than RIPPLELAB in the Zurich dataset, the results were generally comparable between RIPPLELAB and PyHFO in both detectors.

**Table D.1.** HFO classification on new events produced by RIPPLELAB and PyHFO in three datasets.

	STE			MNI		
	Total	Real	Artifact	Total	Real	Artifact
UCLA						
new-RIPPLELAB	602	519	83	773	607	166
new-PyHFO	609	520	89	736	577	159
Zurich						
new-RIPPLELAB	1933	1064	869	5154	2292	2862
new-PyHFO	2059	1994	65	5233	3387	1846
Rodent						
new-RIPPLELAB	5	5	0	3	3	0
new-PyHFO	8	7	1	0	0	0

### D.3. Run-time comparison

Table D.2 shows a comparative analysis of runtime (measured in minutes) in RIPPLELAB and PyHFO: Detection of all events from UCLA, Zurich, and Rodent datasets. When $n > $1 represents when PyHFO runs in a multi-core setup, *n*-jobs = 32 for the Linux machine and *n*-jobs = 8 for macOS and Windows machines.

**Table D.2.** Comparative analysis of runtime (measured in minutes) in RIPPLELAB and PyHFO: Detection of all events from UCLA, Zurich, and Rodent datasets. When $n > $1 represents when PyHFO runs in a multi-core setup, *n*-jobs = 32 for the Linux machine and *n*-jobs = 8 for macOS and Windows machines. The best performance in each machine and dataset was highlighted in bold.

	Linux		Windows		macOS	
	STE	MNI	STE	MNI	STE	MNI
UCLA						
RIPPLELAB	372.83	5647.12	—	—	—	—
PyHFO($n = $1)	57.43	971.35	34.57	933.31	35.90	659.63
PyHFO ($n > $ 1)	**5.18**	**83.30**	**9.03**	**113.59**	**7.56**	**114.35**
Zurich						
RIPPLELAB	468.07	9595.09	—	—	—	—
PyHFO ($n = $ 1)	129.07	2416.18	69.80	1570.95	105.93	1447.27
PyHFO ($n > $ 1)	**22.39**	**324.02**	**21.80**	**320.96**	**31.25**	**237.38**
Rodent						
RIPPLELAB	1.50	49.78	—	—	—	—
PyHFO ($n = $ 1)	0.38	13.93	0.27	8.63	0.35	6.28
PyHFO ($n > $ 1)	**0.1**	**3.3**	**0.13**	**2.52**	**0.15**	**2.00**

### D.4. Events comparison on detecting fast ripple

We further subcategorized the detected events from table 1 into ripple (peak frequency<250 Hz) and fast ripple (peak frequency>250 Hz) and compared the detection results between PyHFO and RIPPLELAB separately. Tables D.3 and D.4 demonstrated the detection comparison. The overall discrepancy was defined as the sum of the number of new HFOs detected by the RIPPLELAB (new-RIPPLELAB) and the number of new HFOs detected by the PyHFO (new-PyHFO) divided by the total number of HFOs detected by the RIPPLELAB. The discrepancies between PyHFO and RIPPLELAB of STE and MNI detector were comparable to when we compared them jointly in table 1. It was important to note that the total discrepancy count (for instance, new-RIPPLELAB_ripple_ + new-RIPPLELAB$_{\text{fast ripple}})$ might be slightly higher than the figures reported in table 1. This variation could occur because certain events with peak frequencies near the threshold (250 Hz) might be classified differently due to minor variations in their start and end times.

**Table D.3.** Ripple events (HFOs with peak frequency<250 Hz, subcategorized from table 1) comparison of differences between RIPPLELAB and PyHFO implementations in UCLA, Zurich, and Rodent dataset. The new-RIPPLELAB row represents the number of new events detected by RIPPLELAB, and the new-PyHFO row represents the number of new events detected by PyHFO for the specific experiment we conducted when the agreement of two events is defined as the 50% overlap of two events. Additionally, the difference between 90% overlap and 50% overlap is minimal, amounting to no more than 0.2% $\left(\frac{n_{\text{overlap 50} \%;}-n_{\text{overlap 90} \%;}}{n_{\text{overlap 90} \%;}}\right)$. (Exp1: data reading and bandpass filter were from RIPPLELAB, but detection algorithm was from PyHFO; Exp2: data reading was from RIPPLELAB, but bandpass filter and detection algorithm were from PyHFO; Exp3: bandpass filter was from RIPPLELAB, but data reading and detection algorithm were from PyHFO; PyHFO: all data reading, bandpass filter and detection algorithm were from PyHFO.)

	No. Events From STE Detector					No. Events From MNI Detector				
	RIPPLELAB	Exp1	Exp2	Exp3	PyHFO	RIPPLELAB	Exp1	Exp2	Exp3	PyHFO
UCLA										
Total Ripples	12 188	12 188	12 115	12 262	12 175	10 098	10 096	10 028	10 125	10 061
Exactly Same	—	12 188	11 179	9142	8694	—	10 076	9730	7562	7346
90% overlap	—	12 188	11 878	11 827	11 605	—	10 096	9823	9516	9321
50% overlap	—	12 188	11 878	11 844	11 620	—	10 096	9823	9513	9328
new-RIPPLELAB	—	0	295	345	553	—	2	258	584	750
new-PyHFO	—	0	237	418	555	—	0	205	612	733
Zurich										
Total Ripples	30 464	30 464	30 465	30 784	30 795	67 084	66 820	66 440	68 169	67 638
Exactly Same	—	30 464	27 439	21 354	19 912	—	66 780	65 063	49 440	48 448
90% overlap	—	30 464	29 638	29 421	28 751	—	66 815	65 524	63 514	62 434
50% overlap	—	30 464	29 638	39 456	28 785	—	66 845	65 526	63 601	62 523
new-RIPPLELAB	—	0	826	1008	1679	—	269	1558	3483	4561
new-PyHFO	—	0	827	1328	2010	—	5	914	4568	5115
Rodent										
Total Ripples	117	117	117	115	115	39	39	36	39	36
Exactly Same	—	117	117	96	96	—	39	36	31	28
90% overlap	—	117	117	113	113	—	39	36	39	36
50% overlap	—	117	117	113	113	—	39	36	39	36
new-RIPPLELAB	—	0	0	4	4	—	0	3	0	3
new-PyHFO	—	0	0	2	2	—	0	0	0	0

**Table D.4.** Fast ripple events (HFOs with peak frequency≥250 Hz, subcategorized from table 1) comparison of differences between RIPPLELAB and PyHFO implementations in UCLA, Zurich, and Rodent dataset. The new-RIPPLELAB row represents the number of new events detected by RIPPLELAB, and the new-PyHFO row represents the number of new events detected by PyHFO for the specific experiment we conducted when the agreement of two events is defined as the 50% overlap of two events. Additionally, the difference between 90% overlap and 50% overlap is minimal, amounting to no more than 0.2% $\left(\frac{n_{\text{overlap 50} \%;}\unicode{x2014}n_{\text{overlap 90} \%;}}{n_{\text{overlap 90} \%;}}\right)$.(Exp1: data reading and bandpass filter were from RIPPLELAB, but detection algorithm was from PyHFO; Exp2: data reading was from RIPPLELAB, but bandpass filter and detection algorithm were from PyHFO; Exp3: bandpass filter was from RIPPLELAB, but data reading and detection algorithm were from PyHFO; PyHFO: all data reading, bandpass filter and detection algorithm were from PyHFO.

	No. Events from STE detector					No. Events from MNI detector				
	RIPPLELAB	Exp1	Exp2	Exp3	PyHFO	RIPPLELAB	Exp1	Exp2	Exp3	PyHFO
UCLA										
Total HFO	306	306	320	320	326	294	294	292	297	294
Exactly same	—	306	274	252	228	—	294	290	208	207
90% overlap	—	306	292	299	288	—	294	290	263	260
50% overlap	—	306	292	299	288	—	294	290	263	260
new-RIPPLELAB	—	0	14	7	18	—	0	2	31	34
new-PyHFO	—	0	14	21	38	—	0	2	34	34
Zurich										
Total HFO	1280	1280	1062	1305	1074	3904	3718	3320	3825	3429
Exactly same	—	1280	900	1061	791	—	3716	3292	2272	2545
90% overlap	—	1280	1001	1230	970	—	3717	3313	3370	3040
50% overlap	—	1280	1001	1230	970	—	3717	3313	3372	3041
new-RIPPLELAB	—	0	279	50	301	—	187	591	532	863
new-PyHFO	—	0	61	75	104	—	1	7	453	388
Rodent										
Total HFO	258	258	258	263	263	3	3	3	3	3
Exactly same	—	258	257	228	228	—	3	3	3	3
90% overlap	—	258	258	255	255	—	3	3	3	3
50% overlap	—	258	258	255	255	—	3	3	3	3
new-RIPPLELAB	—	0	0	3	3	—	0	0	0	0
new-PyHFO	—	0	0	8	8	—	0	0	0	0
